# Synergistic Pyro‐Phototronics and Structural Anisotropy in CsAg_2_I_3_/GaN Heterostructures for High‐Performance Polarization‐Sensitive UV Photodetectors

**DOI:** 10.1002/advs.74896

**Published:** 2026-03-18

**Authors:** Yalin Zhai, Siqi Wang, Peng Guo, Songling Feng, Peng Wan, Jilong Tang, Caixia Kan, Hui Li, Daning Shi, Mingming Jiang

**Affiliations:** ^1^ College of Physics MIIT Key Laboratory of Aerospace Information Sensing and Physics Key Laboratory for Intelligent Nano Materials and Devices Nanjing University of Aeronautics and Astronautics Nanjing Jiangsu P. R. China; ^2^ Jilin Key Laboratory of Solid‐State Laser Technology and Application School of Physics Changchun University of Science and Technology Changchun Jilin P. R. China; ^3^ State Key Laboratory of High Power Semiconductor Laser Changchun University of Science and Technology Changchun Jilin P. R. China

**Keywords:** CsAg_2_I_3_ single‐crystals, information‐splitting encryption, polarization‐sensitive photodetection, pyro‐phototronic effect, van der Waals heterojunction

## Abstract

Ultraviolet photodetectors capable of spectral selectivity, self‐powered operation, and polarization sensitivity are vital for next‐generation secure communication and sensing, yet their progress is hampered by material instability, interfacial losses, and the difficulty of multifunctional integration. Here, we report a groundbreaking CsAg_2_I_3_/GaN van der Waals (vdWs) heterojunction that uniquely integrates in‐plane structural anisotropy, pronounced pyro‐phototronic effect, and atomically sharp interface engineering. The CsAg_2_I_3_ single‐crystals, synthesized via chemical vapor deposition, exhibit a wide‐bandgap (∼3.38 eV), strong second‐order nonlinear response, and remarkable pyroelectricity, overcoming the stability and symmetry limitations of conventional perovskites and inorganic wide‐bandgap semiconductors. The CsAg_2_I_3_/GaN device delivers record‐breaking performance: an ultralow dark current of 0.3 pA, high responsivity of 0.28 A/W, specific detectivity of 1.7  × 10^12^ Jones, and ultrafast response speeds of 16/21 µs, outperforming most state‐of‐the‐art perovskites and wide‐bandgap photodetectors. Crucially, the pyro‐phototronic effect amplifies polarization sensitivity, achieving an unprecedented dichroic ratio of 10.5 under bias, the highest reported for all‐inorganic perovskite systems and other competitors. The device also demonstrates exceptional operational stability and robust performance in an information‐splitting encryption system, validating its real‐world applicability. This work establishes a new paradigm for synergizing pyro‐phototronics and structural anisotropy in heterostructures, enabling a versatile platform for high‐performance, self‐powered, polarization‐sensitive optoelectronics.

## Graphical abstract

The development of polarization‐sensitive ultraviolet photodetectors is limited by poor heterojunction quality and low polarization sensitivity. This study integrates synthesized CsAg_2_I_3_ single crystals with intrinsic non‐centrosymmetry into van der Waals heterojunction devices, demonstrating pronounced pyro‐phototronic effect. High dichroic ratio is validated via information‐splitting encryption system, establishing a novel paradigm for intelligent and secure optoelectronic technologies.

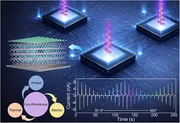

## 1 Introduction

The development of high‐quality, wide‐bandgap single crystals (SCs) that possess intrinsic non‐centrosymmetric structures and exhibit strong pyro‐phototronic responses constitutes the fundamental cornerstone for the advancement of miniaturized and self‐powered optoelectronic systems [1, 2, 3]. This unique combination of properties is crucial for the detection of polarized light and efficient pyro‐phototronic energy conversion, which are indispensable in advanced ultraviolet (UV) photonics, polarimetric imaging, and secure communication [4]. Existing wide‐bandgap semiconductors, such as Ga_2_O_3_, AlGaN, and others, typically require external symmetry‐breaking interventions like doping or strain engineering [5, 6, 7, 8]. The inherent complexity of these methodologies can deleteriously affect the materials' optoelectronic characteristics, leading to a significant reduction in two key figures of merit: carrier mobility and optical transparency [9]. Lead‐free metal halide perovskites, characterized by long carrier diffusion lengths, high light absorption coefficients, low defect densities, and environmental friendliness, have been leveraged for polarization‐sensitive photodetectors (PDs) due to the anisotropy of their crystalline structures and the controllability of oriented growth [10, 11, 12, 13, 14]. A critical bottleneck for the field is the inherent instability of many non‐centrosymmetric perovskites that otherwise exhibit exceptional pyroelectric performance. Their tendency to decompose and facilitate ionic transport, even under mild operating conditions, leads to unrelenting degradation, posing a seemingly intractable challenge for achieving commercially viable operational lifespans [15, 16, 17]. Therefore, the design and synthesis of novel stable halide perovskites featuring wide bandgaps, intrinsic structural asymmetry, and pronounced pyro‐phototronic coefficients are of critical importance for realizing high‐performance, low‐power‐consumption, and integrable UV PDs.

The full potential of wide‐bandgap semiconductors with structural asymmetry can only be realized through high‐quality heterojunctions, a task that faces two major challenges [18, 19]. First, interfacial defect states act as efficient recombination centers, significantly weakening the built‐in electric field, thereby drastically reducing the self‐powered photovoltaic and pyroelectric responses. Second, due to the limited chemical tunability of these materials, achieving the precise type‐II or Z‐scheme band alignments required for efficient carrier separation is extremely challenging [20, 21, 22]. Van der Waals (vdWs) integration offers a transformative solution by enabling atomically sharp interfaces, thereby circumventing lattice mismatch and minimizing trap states [23]. The pyro‐phototronic effect, which couples light, heat, and electricity, can comprehensively enhance device performance metrics by modulating the processes of carrier generation, separation, and transport [24, 25, 26]. The ultimate challenge and opportunity lie in achieving an organic integration of steady‐state photovoltaic effects, transient pyro‐phototronic responses, and the inherent structural anisotropy of materials, thereby injecting new vitality into self‐powered UV photonics and optoelectronic systems.

In this study, we address these challenges through a concerted strategy integrating crystal growth and heterojunction engineering. First, the sample of high‐quality, layered‐like CsAg_2_I_3_ SCs with an intrinsic non‐centrosymmetric structure was synthesized via an optimized chemical vapor deposition (CVD) method, which mitigates the high defect density and instability prevalent in solution‐grown crystals. The obtained crystals exhibit strong in‐plane optical anisotropy, a pronounced second‐order nonlinear optical response, and a significant pyro‐phototronic characteristic. Second, by integrating these crystals with GaN substrate, we innovatively fabricated a high‐quality vdWs heterojunction self‐powered, polarization‐sensitive UVA PD, achieving an ultralow dark current of 0.3 pA and an on/off ratio of 10^4^. The device performance is profoundly enhanced by the synergistic pyro‐phototronic effect at the heterointerface, achieving a peak responsivity of 0.28 A/W, a high specific detectivity of 1.7 × 10^12^ Jones, ultrafast response speeds of 16/21 µs, and an external quantum efficiency exceeding 95% under 365 nm illumination. Remarkably, this effect substantially amplifies polarization discrimination, yielding an exceptionally high dichroic ratio that reaches a record‐breaking value of 10.5 at −2 V. We further demonstrate the practical potential of this device in information‐splitting encryption for secure optical communication. Our work not only elucidates the operational principles of polarization‐sensitive PDs based on asymmetric crystal vdW heterostructures but also provides a design blueprint for future intelligent, self‐powered optoelectronic systems.

## 2 Results and Discussion

**FIGURE 1:**
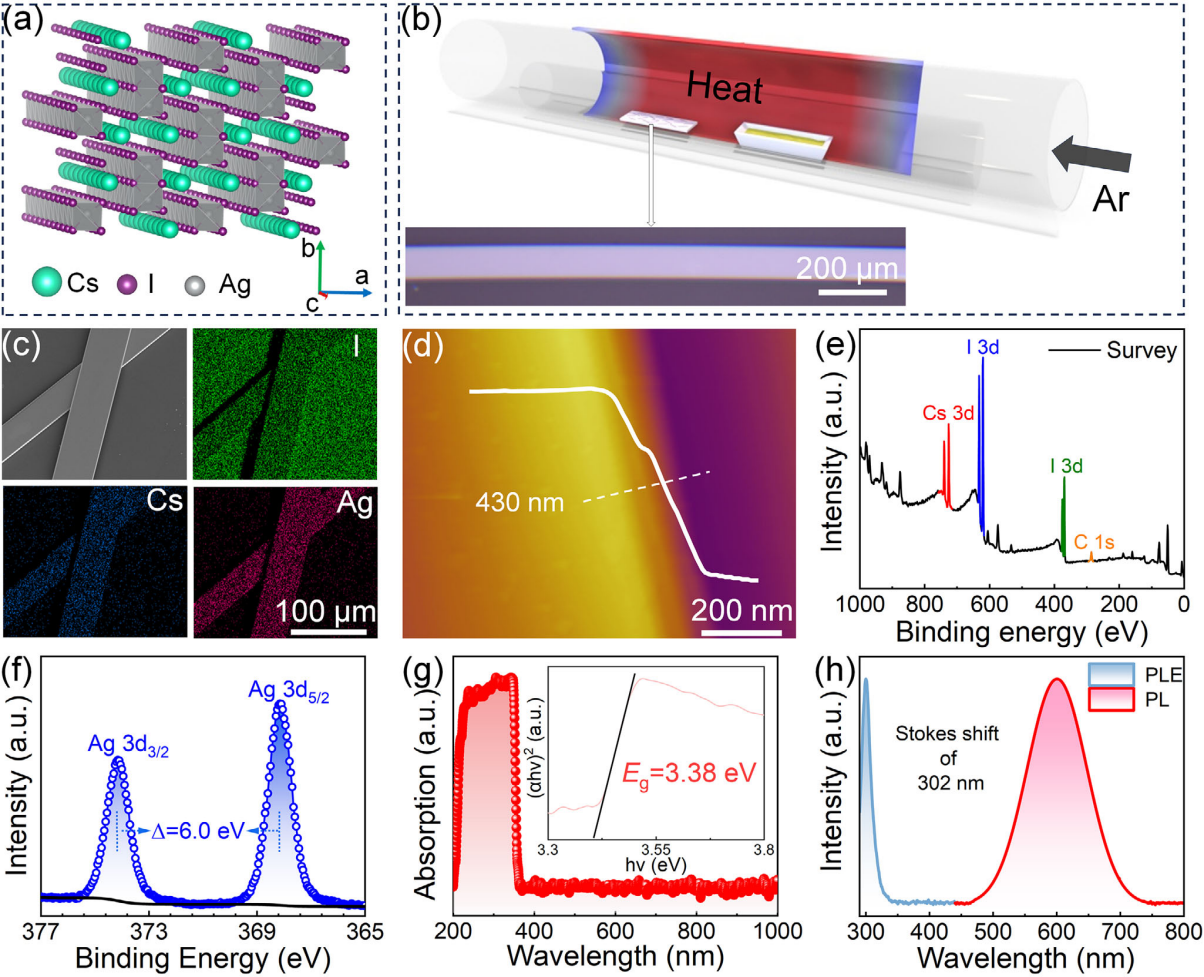
Synthesis and characterization of the CsAg_2_I_3_ perovskite. (a) Sketch of the crystal structure of CsAg_2_I_3_. (b) Schematic diagram of CsAg_2_I_3_ growth via CVD. (c) SEM and elemental mappings of the CsAg_2_I_3_ SC. (d) AFM and height measured on account of a ribbon‐like CsAg_2_I_3_ SC. (e) XPS survey spectrum of the CsAg_2_I_3_ sample. (f) Ag 3d XPS spectrum of the CsAg_2_I_3_ SC. (g) Absorption spectrum and extracted Tauc plot (upright inset) of the CsAg_2_I_3_ SC. (h) PLE and PL spectra.

Figure 1a shows the 1D wirelike crystal structure of CsAg_2_I_3_ (with unit cell parameters: *a* = 1.37432 nm, *b* = 6.2306 nm, *c* = 1.10763 nm, *α* = *β* = *γ* = 90°), which belongs to the orthorhombic structure. The lattice comprises 1D [AgI_4_]^3−^ tetrahedral chains interconnected along the [001] direction, with adjacent Cs atoms separating these chains. The structure forms an approximate layered arrangement through layer‐by‐layer stacking, as illustrated in Figure S1. This quasi‐2D anisotropic crystal structure gives rise to distinct spatial charge density distributions across different crystalline phases, resulting in varied responses to polarized light in different directions. In this experiment, the process of synthesizing high‐quality CsAg_2_I_3_ SCs via the CVD method is depicted in Figure 1b. Optical microscopy reveals that the synthesized millimeter‐scale ribbon‐like CsAg_2_I_3_ exhibits a colorless and translucent appearance under ambient conditions. The detailed synthesis process is available in the Experimental Section and Table S1. Interestingly, by adjusting the experimental conditions, the width of quasi‐2D CsAg_2_I_3_ SCs can be controllably tuned within the range of 10–100 µm, with the corresponding size distribution profile shown in Figure S2. Furthermore, scanning electron microscope (SEM) images demonstrate that those SCs with different widths are presented in Figure S3.

Under experimental condition 4, a detailed investigation was conducted on the physical and chemical properties of CsAg_2_I_3_ SCs. In Figure 1c, the SEM image of CsAg_2_I_3_ reveals a smooth surface (with a width of approximately 50 µm), indicating favorable carrier transport properties. The corresponding energy dispersive spectroscopy (EDS) mapping demonstrates a uniform distribution of I, Ag, and Cs elements throughout the entire material, indicating its high crystallinity. Furthermore, in the quantitative EDS measurements presented in Figure S4, the quantitative atomic ratio (%) of Cs:Ag:I is determined to be 1:2:3, which closely aligns with the stoichiometry of CsAg_2_I_3_. The atomic force microscopy (AFM) image presented in Figure 1d reveals that the SC exhibits a thickness of approximately 430 nm, with no discernible grain boundaries. High‐resolution transmission electron microscopy (HRTEM) images and the X‐ray diffraction (XRD) patterns presented in Figures S5 and S6 also demonstrate that the CVD‐grown SCs exhibit high phase purity. The chemical properties and valence states of CsAg_2_I_3_ SCs were analyzed using X‐ray photoelectron spectroscopy (XPS). The XPS spectra are depicted in Figure 1e, where the position of the C 1s peak was employed for binding energy calibration. This spectral analysis clearly reveals the composition and bonding states of Ag 3d, Cs 3d, and I 3d within the sample. As Figure 1f shows, the Ag 3d consists of doublet peaks with binding energies at 368.1 eV (3d_5/2_) and 374.1 eV (3d_3/2_), which is consistent with Ag^+^ [12]. Additionally, the Cs and I core‐level spectrum are shown in Figure S7.

Subsequently, the photophysical properties of quasi‐2D CsAg_2_I_3_ SCs were systematically investigated. The micro‐UV–vis absorption spectrum presented in Figure 1g reveals that CsAg_2_I_3_ exhibits a sharp absorption edge at approximately 365 nm. This lead‐free CsAg_2_I_3_ perovskite with strong UV absorption demonstrates significant potential as a promising candidate for UV photodetection applications. The corresponding tac diagram (inset in Figure 1g) indicates that the CVD‐obtained CsAg_2_I_3_ SCs exhibit a wide direct bandgap of 3.38 eV, which is larger than the bandgap obtained through theoretical calculations, as shown in Figure S8. Figure 1h presents the photoluminescence excitation (PLE) and photoluminescence (PL) spectra of CsAg_2_I_3_. The PLE spectrum shows that the highest excitation efficiency occurs at 300 nm, while the PL spectrum exhibits a peak centered at 602 nm with a full width at half maximum of 110 nm. It is generally accepted that broadband emission in metal halides typically originates from self‐trapped excitons (STEs) [27]. Additionally, we observed a substantial Stokes shift of 302 nm (2.08 eV), which represents a common characteristic of low‐dimensional metal halide perovskites exhibiting STEs. Additionally, the PL decay curve presented in Figure S9 shows an average PL lifetime of 2.8 ns, which is relatively short and can be advantageous for the fast response of UV PDs from an application perspective [28].

**FIGURE 2:**
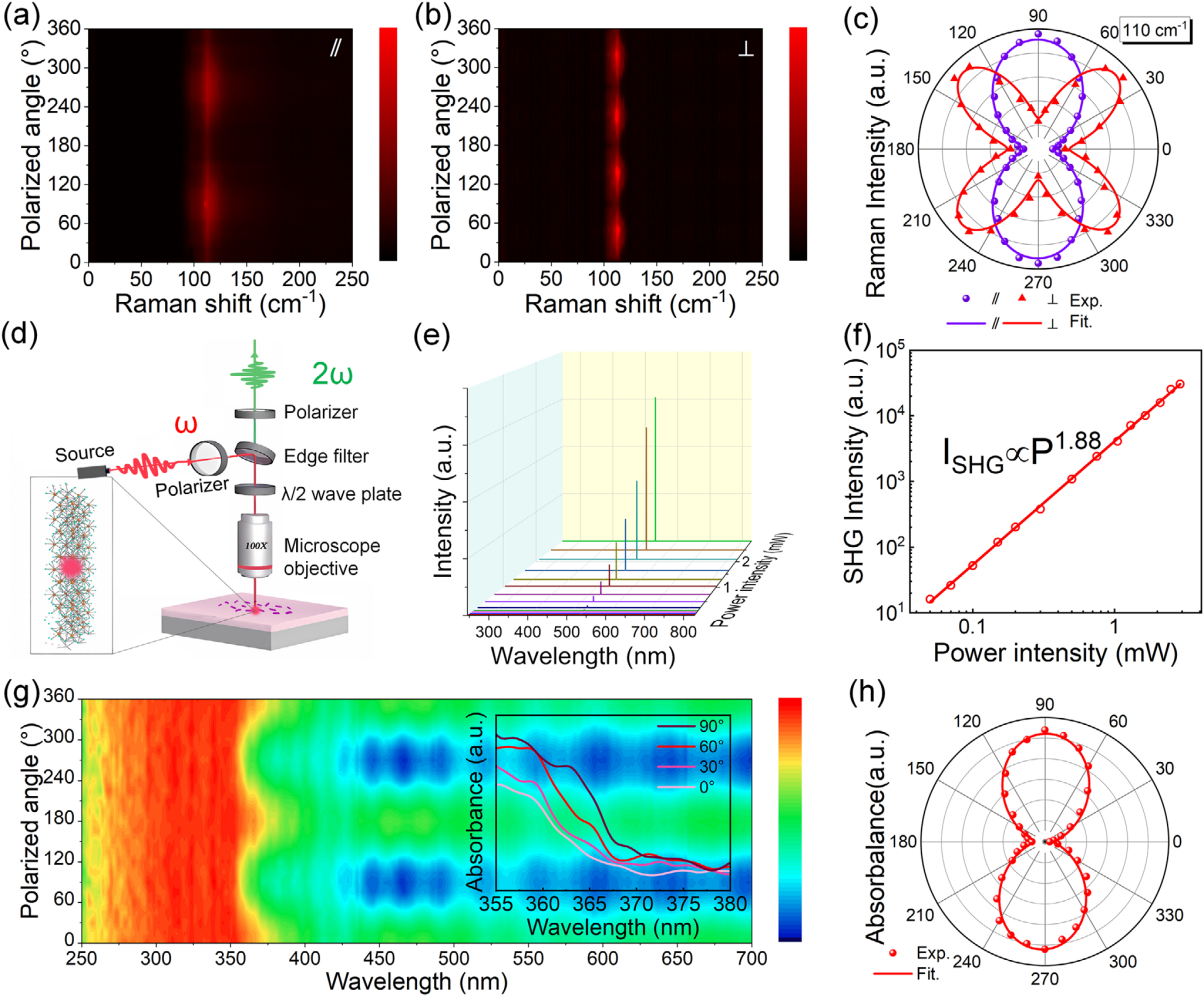
Optical anisotropy of CVD‐synthesized CsAg_2_I_3_ SCs. Contour maps of angle‐resolved Raman spectra under (a) parallel, and (b) perpendicular. (c) Polar plots of angle‐resolved and fitted peak intensity. (d) Schematic of an SHG equipment. (e) Power‐dependent SHG intensity of the CsAg_2_I_3_ SC. (f) The linear fitting of SHG intensity depending in a logarithmic manner. (g) Contour map of polarization‐dependent absorption intensity of the CsAg_2_I_3_ SC. Inset: Angle‐absorption intensity curves from 355 to 380 nm. (h) Polar plots of absorbance at 365 nm.

Raman spectroscopy enables the identification of crystal orientation and evaluation of vibrational symmetry in chemical bonds. Angle‐resolved polarized Raman spectroscopy effectively facilitates its application in probing material anisotropy. Herein, polarized Raman experiments on CsAg_2_I_3_ SCs were conducted using a backscattering configuration with a 532 nm laser. Figure 2a,b displays 2D heatmaps of wavenumber‐dependent Raman intensities across angles ranging from 0° to 360°, measured under parallel and perpendicular configurations, respectively. As evident from these plots, the vibrational intensities of phonon modes, particularly the I‐Ag‐I mode at approximately 110 cm^−1^, exhibit a clear periodicity with changes in the polarization angle in the parallel (perpendicular) configuration, with a modulation amplitude of 180° (90°). In Figure 2c, the variation of Raman peak intensity with polarization angle was further extracted and plotted in a polar coordinate system. The fitting curve of Raman intensity is determined by the following formula: [29]

(1) $$I\propto {\left[e_{i}\cdot R\cdot e_{s}\right]}^{2}$$

where *I* is the Raman intensity, *e*_i_ is the polarization vector of incident light (*e*_i_ = [0 cos*θ* sin*θ*], *θ* is the polarization angle determined by rotating the half‐wave plate by *θ*/2), *e*_s_ is the polarization vector of scattered light (*e*_s//_ = [0 cos*θ* sin*θ*]*^T^*, *e*_s⊥_ = [0 ‐sin*θ* cos*θ*]*^T^*), and *R* is the Raman tensor. The Raman tensors of A_g_ (CsAg_2_I_3_ corresponds to D_2h_ symmetry groups) appear as: [30]

(2) $$A_{g}=\left[\begin{array}{ccc} a & 0 & 0\\ 0 & b & 0\\ 0 & 0 & c \end{array}\right]$$

After matrix operations, the formula for the fitted curve can be expressed in the following form:

(3) $$I\left(A_{g//}\right)\propto {\left|b\cdot \cos^{2}\theta +c\cdot \sin^{2}\theta \right|}^{2}$$

(4) $$I\left(A_{g⊥}\right)\propto \frac{{\left|a-b\right|}^{2}}{4}\sin^{2}2\theta$$

The calculated vibrational mode undoubtedly exhibits polarization‐sensitive characteristics, as evidenced by the highly correlated Raman intensity curves. In the parallel polarization configuration, the intensity exhibits a bimodal distribution, whereas a quadrimodal pattern is observed in the perpendicular configuration. These findings corroborate the pronounced in‐plane structural anisotropy of the quasi‐2D CsAg_2_I_3_ SCs.

To investigate the nonlinear optical and polarization‐dependent optical properties of ribbon‐like CsAg_2_I_3_ SCs, a 1064 nm pulsed laser was employed as the pump source to study their second harmonic generation (SHG) performance. The detailed experimental setup is illustrated in Figure 2d. Figure 2e displays the SHG spectrum of CsAg_2_I_3_ SCs under varying laser irradiation intensities, exhibiting a characteristic peak at 532 nm. This spectral feature originates from the SHG process of the excitation laser, which arises from nonlinear optical interactions between incident photons and the non‐centrosymmetric crystal lattice. By fitting the relationship between SHG intensity and incident laser power, a power exponent of 1.88 was obtained as shown in Figure 2f, thereby confirming the order of the underlying nonlinear process [31]. For CsAg_2_I_3_ crystals belonging to a centrosymmetric space group, the observed SHG can be attributed to the breaking of internal symmetry. In the field of photodetection, such non‐centrosymmetric crystal structures with spontaneous polarization, as seen in materials like ZnO and BaTiO_3_, can induce the pyro‐phototronic effect, significantly enhancing the performance of PDs [32]. Thermal imaging measurements were performed on SCs under UV illumination (λ = 365 nm, power density = 5.0 mW/cm^2^), as shown in Figure S10. To ensure that the measured thermal effects originate exclusively from the sample, the thermal contributions from the glass substrate and background interference have been excluded, as demonstrated in Figure S11. The thermal imaging results reveal that CsAg_2_I_3_ can autonomously absorb UV light and generate heat under weak illumination, exhibiting significant temperature variations. Furthermore, the polarization diagrams show the angular‐resolved SHG intensity measured in both parallel and perpendicular configurations, as depicted in Figure S12. The anisotropic atomic arrangement along distinct crystallographic directions affects the probability of interaction between incident photons and atoms. Certain directions exhibit a higher probability of SHG, manifesting as larger lobes in the polar plot, while smaller lobes are formed in other directions. The observed pronounced directional dependence, attributed to planar anisotropy associated with symmetry breaking, provides compelling evidence for the high anisotropy of the CsAg_2_I_3_ crystal structure [12].

The asymmetric crystal structure of CsAg_2_I_3_ facilitates efficient photon absorption. Under such conditions, the polarized angle‐dependent optical absorption in CsAg_2_I_3_ SCs was measured using a micro‐absorption apparatus (Figure S13). The incident light signal was collected through the crystal and an ultra‐thin quartz substrate, with its initial polarization direction parallel to the long side of the crystal. In the polarization‐dependent absorption spectra presented in Figure 2g, the sample was tested under a parallel polarization configuration, revealing significant absorption variations with incident polarization angle and exhibiting 180° periodicity in the UV spectral range. A significant displacement of the absorption edge is observed as the polarization angle of the incident light varies. Figure 2h illustrates the polarization‐angle‐dependent variation of absorbance in a polar coordinate plot. The in‐plane anisotropy of optical absorption in CsAg_2_I_3_ reflects intrinsic refractive index variations across different crystal orientations.

**FIGURE 3:**
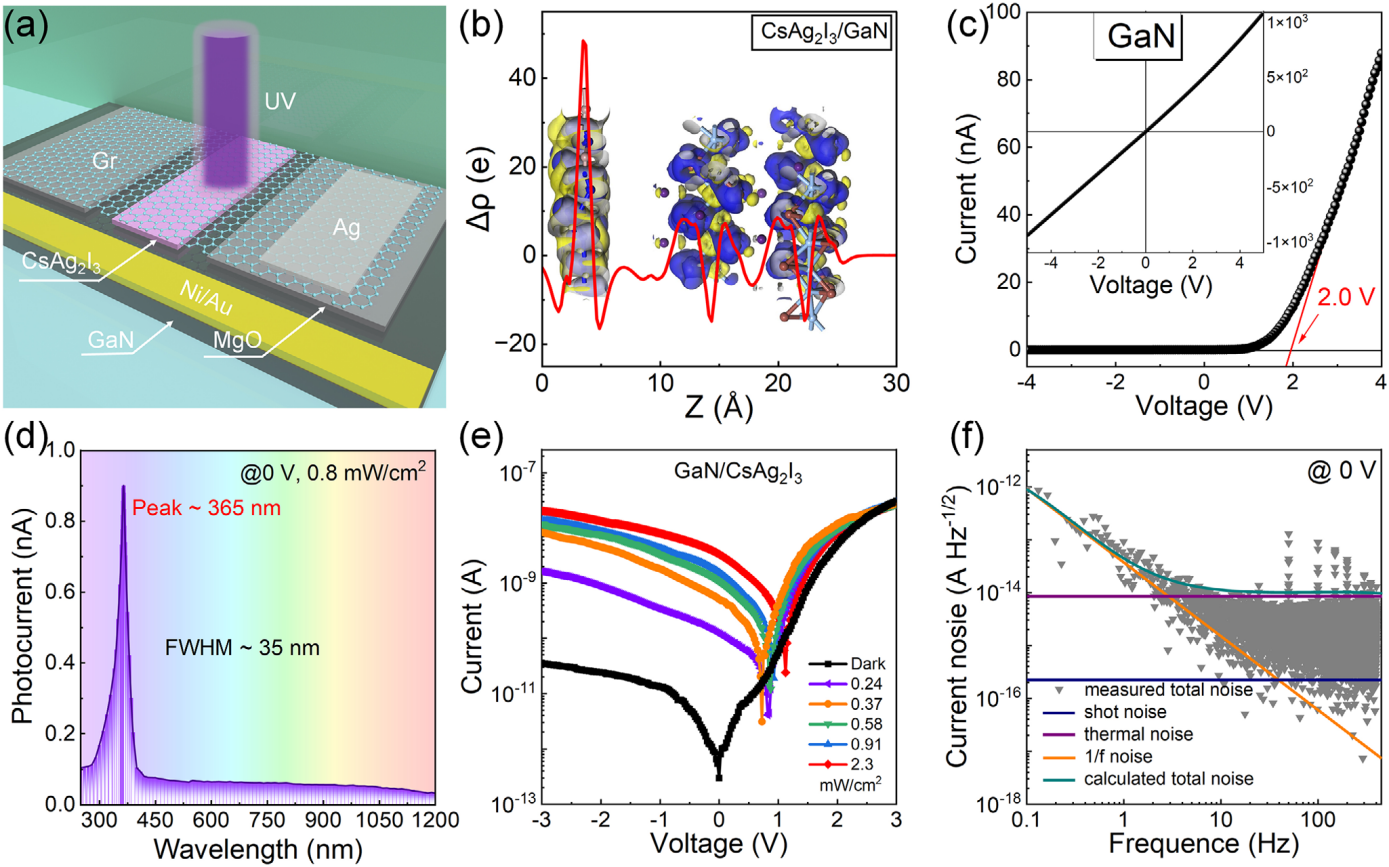
Photoresponse characteristics of the CsAg_2_I_3_/GaN vdWs heterojunction PD. (a) The schematic diagram of the device. (b) The differential charge density of the CsAg_2_I_3_/GaN interface. (c) *I–V* curve of the device in the dark. The inset shows the *I–V* curve of the GaN template. (d) Wavelength‐dependent photocurrent of the PD at zero bias. (e) Light irradiance‐dependent *I–V* curves under 365 nm illumination. (f) Total noise power density spectrum obtained by the Fourier transform of the time‐domain dark current.

CsAg_2_I_3_ exhibits exceptional properties, including a wide bandgap, ultrafast lifetime, asymmetric crystal structure, and anisotropic optical absorption, which collectively render it an ideal candidate for constructing polarization‐sensitive UV PDs. A metal‐semiconductor‐metal (MSM) PD was fabricated by thermally evaporating symmetric gold electrodes, with its photoresponse characteristics presented in Figure S14. However, the suboptimal photoresponse and low polarization sensitivity of this configuration fail to meet practical application requirements. Developing heterojunction‐based PDs may offer an effective pathway toward high‐performance device fabrication. As shown in Figure 3a, the flake‐shaped CsAg_2_I_3_ SC was transferred onto pre‐patterned MgO trenches, forming vdWs contacts with GaN (N‐type electrical conduction). Subsequently, a single‐layer graphene (Gr) with high optical transmittance was transferred to serve as the top electrode, with its transmittance spectrum presented in Figure S15. Through differential charge density calculations, the interface charge transfer pathway of the CsAg_2_I_3_/GaN structure is further elucidated. As illustrated in Figure 3b, the differential charge density map displays regions colored yellow and blue, representing electron loss and gain, respectively. The results reveal the presence of multiple charge accumulation and depletion regions near the CsAg_2_I_3_/GaN interface, indicating active charge transfer between the CsAg_2_I_3_ surface and GaN. Notably, a strong charge accumulation region is observed on the GaN surface adjacent to the interface, confirming the process of charge transfer from CsAg_2_I_3_ to GaN and suggesting that this vdWs heterostructure possesses favorable charge transfer capabilities in theory.

The *I–V* curve of the CsAg_2_I_3_/GaN vdWs heterojunction, as prepared and shown in Figure 3c, exhibits typical diode rectification characteristics with a large open‐circuit voltage of 2.0 V and an ultralow dark current of 0.3 pA. The inset displays a representative *I–V* curve measured for a Ni/Au‐GaN‐Ni/Au structure using a two‐probe system. The *I–V* curve of the GaN thin film measured under dark conditions exhibits linearity, indicating high‐quality ohmic contact between the Ni/Au electrodes and the N‐GaN layer. As shown in Figure 3d, broadband photocurrent measurements were conducted across the 250–1200 nm wavelength range under identical optical power conditions, with a peak at 365 nm. Interestingly, a measurable photoresponse was still observed when the material was illuminated with wavelengths beyond the absorption edge. The *I–t* curves under visible and near‐infrared illumination, as presented in Figure S16, indicate a strong pyroelectric current, which is consistent with the previously mentioned heat accumulation in CsAg_2_I_3_ upon UV absorption. As shown in Figure 3e, by varying the incident 365 nm light irradiance from 0.24 to 2.3 mW/cm^2^, the photocurrent at 0 V bias gradually increased. Energy band diagram of the self‐powered CsAg_2_I_3_/GaN vdWs heterojunction PD under UV illumination, as shown in Figure S17. The total noise (*i_n_*) of the vdWs heterojunction PD, which is influenced by shot noise (*i*_shot_), flicker 1/*f* noise (*i*_1/_ *_f_*), and thermal noise (*i*_thermal_), is analyzed at zero bias as depicted in Figure 3f. They are calculated by: [4]

(5) $$\langle i_{shot}^{2}\rangle =2qI_{d}\Delta f$$

(6) $$\langle i_{1/f}^{2}\rangle =CI_{d}^{\beta }/f^{\alpha }\Delta f$$

(7) $$\langle i_{thermal}^{2}\rangle =4kT\Delta f/R_{s}$$

(8) $$\langle i_{n}^{2}\rangle =\langle i_{shot}^{2}\rangle +\langle i_{1/f}^{2}\rangle +\langle i_{thermal}^{2}\rangle$$

where *I*_d_ is the dark current, *f* is the frequency, *Δf* is the electronic bandwidth, and *R*s is the shunt resistance. *C*, *α*, and *β* are constants. The measured total noise acquired from a rapid Fourier transform of the time‐domain dark current is perfectly fitted with the calculated total noise. At frequencies below 10 Hz, flicker *i_1/f_* noise is mostly caused by scattering at the CsAg_2_I_3_ SC and the interface contact. The low flicker *i_1/f_* noise further corroborates that the vdWs junction formed between the CsAg_2_I_3_ SC and GaN is highly optimized, enabling the device to exhibit superior detection capability. The high‐frequency noise is primarily determined by thermal noise, which is mainly caused by the thermal transport of charge carriers.

**FIGURE 4:**
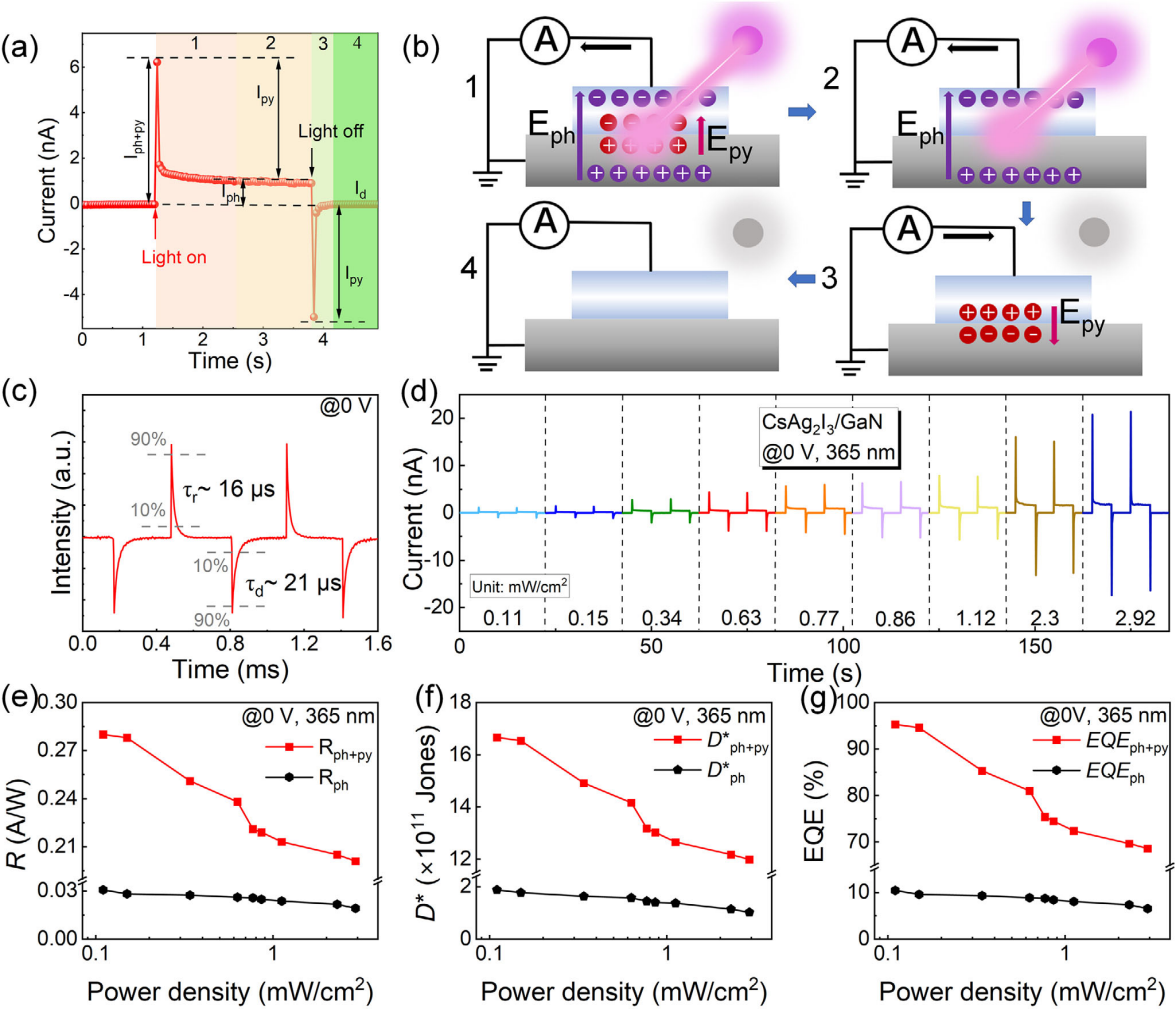
Pyro‐phototronic effect enhanced UV photodetection performances of the PD. (a) The characteristic transient photocurrent response generated by the self‐powered PD under the pyro‐phototronic effect (365 nm, 0.8 mW/cm^2^). (b) Schematic representation of the photoelectric response mechanism in the CsAg_2_I_3_/GaN vdWs heterojunction PD corresponding to the four regions depicted in (a). (c) Time‐resolved photoresponse of the device. (d) *I–t* characteristics of the PD under 365 nm illumination with different power densities. (e) *R*_ph+py_ and *R*_ph_ as a function of power density. (f) D^*^_ph+py_ and D^*^_ph_ as a function of power density. (e) *EQE*_ph+py_ and *EQE*_ph_ as a function of power density.

To systematically investigate the pyro‐phototronic effect in the PD, a magnified segment of the *I–t* curve is presented in Figure 4a, wherein the current variations are categorized into four distinct regions (region 1, region 2, region 3, and region 4). In region 1, the activation of the UV lamp induces a rapid photothermal effect, generating thermally activated charges that modulate the local potential and effectively lower the heterojunction interface barrier. This barrier reduction facilitates more efficient separation and transport of photoinduced charge carriers. In this regard, a sharp peak appears in the photocurrent, which is labeled as *I*_ph+py_. After a certain period, the temperature of the CsAg_2_I_3_ SC stabilizes, and the pyroelectric effect vanishes. In this case, this response current in region 2 decays to *I*_ph_, which can be attributed to the photovoltaic effect in the device [33]. Upon the extinction of UV illumination, the abrupt temperature drop induces a reverse distribution of pyroelectric charges across the CsAg_2_I_3_ SC, as depicted in Figure 4b, thereby generating a reverse pyroelectric current in region 3. Finally, in region 4, the response current approaches the dark current (*I*_d_) as the temperature stabilizes in the absence of UV illumination. This is a typical dynamic current behavior induced by the pyro‐phototronic effect [26].

Response speed, characterized by the rise and fall times, serves as a decisive performance metric for PDs. The rise time (*τ*_r_) is defined as the interval required for the optical response to transition from 10% to 90% of its peak value, whereas the fall time (*τ*_d_) corresponds to the reverse transition from 90% to 10%. Harnessing the power of the pyro‐phototronic effect, this detector achieves an exceptionally high response speed. Critically, the transient photoresponse, depicted in Figure 4c, features rapid rise and fall times of 16 and 21 µs under a 365 nm light irradiance of 0.2 mW/cm^2^. This performance benchmark represents a significant advancement, surpassing the metrics of comparable wide‐bandgap semiconductor photodetection devices detailed in Table S2. A systematic study of the dynamic response of the fabricated CsAg_2_I_3_/GaN vdWs heterojunction device under 365 nm illumination was conducted by varying the power density from 0.11 to 2.92 mW/cm^2^, and the results are summarized in Figure 4d. It is evident that, under zero bias, a four‐region current dynamic behavior induced by the pyro‐phototronic effect is observed at all power densities. The pyro‐phototronic signal remains stable across multiple consecutive cycles, demonstrating the device's robust stability as evidenced in Figure S18. Then, by fitting the data through the power law of *I* = *α*·*P^β^*, where *α* is a constant, and *β* is an exponent, a value of *β* = 0.9 is derived (Figure S19), close to that of an ideal detector (*β* = 1) [34]. Additionally, the key photoresponse metrics, such as photoresponsivity (*R*), specific detectivity (D*), and external quantum efficiency (*EQE*), are evaluated as: [4]

(9) $$R=\left(I-I_{d}\right)/PS$$

(10) $$D^{*}=R\sqrt{S\Delta f}/I_{n}$$

(11) EQE=Rhc/eλ

The variable *I*_d_, *I*_n_, *P*, and *S* correspond to dark current, current noise (2.2 × 10^−14^ A Hz^−1/2^ at 1 Hz), power density, and the active area (*S* ∼ 3400 µm^2^), respectively. Meanwhile, *c*, *e*, and *h* represent the speed of light in vacuum, the elementary charge, and Planck's constant. As shown in Figure 4e–g, the transient pyroelectric current (*I*_py_) induced by UV illumination significantly enhances the detector performance, achieving a high *R* of 0.28 A/W, a remarkable *D* ^*^of 1.7 × 10^12^ Jones, and an exceptional *EQE* of 95.3% under the low optical power condition. The presence of the pyro‐phototronic effect comprehensively enhances the optoelectronic performance of the device, as depicted in Figure S20. The CsAg_2_I_3_/GaN vdWs heterojunction detector demonstrated here achieves an unprecedented level of performance, positioning it as a highly competitive candidate among leading wide‐bandgap semiconductor PDs. It not only outperforms all previously reported Ag/Cu‐based perovskite devices but also rivals, and in key metrics surpasses, established inorganic wide‐bandgap technologies. Moreover, after being stored indoors for 15 weeks, the device's performance exhibited less than a 6% change from its initial values (Figure S21).

**FIGURE 5:**
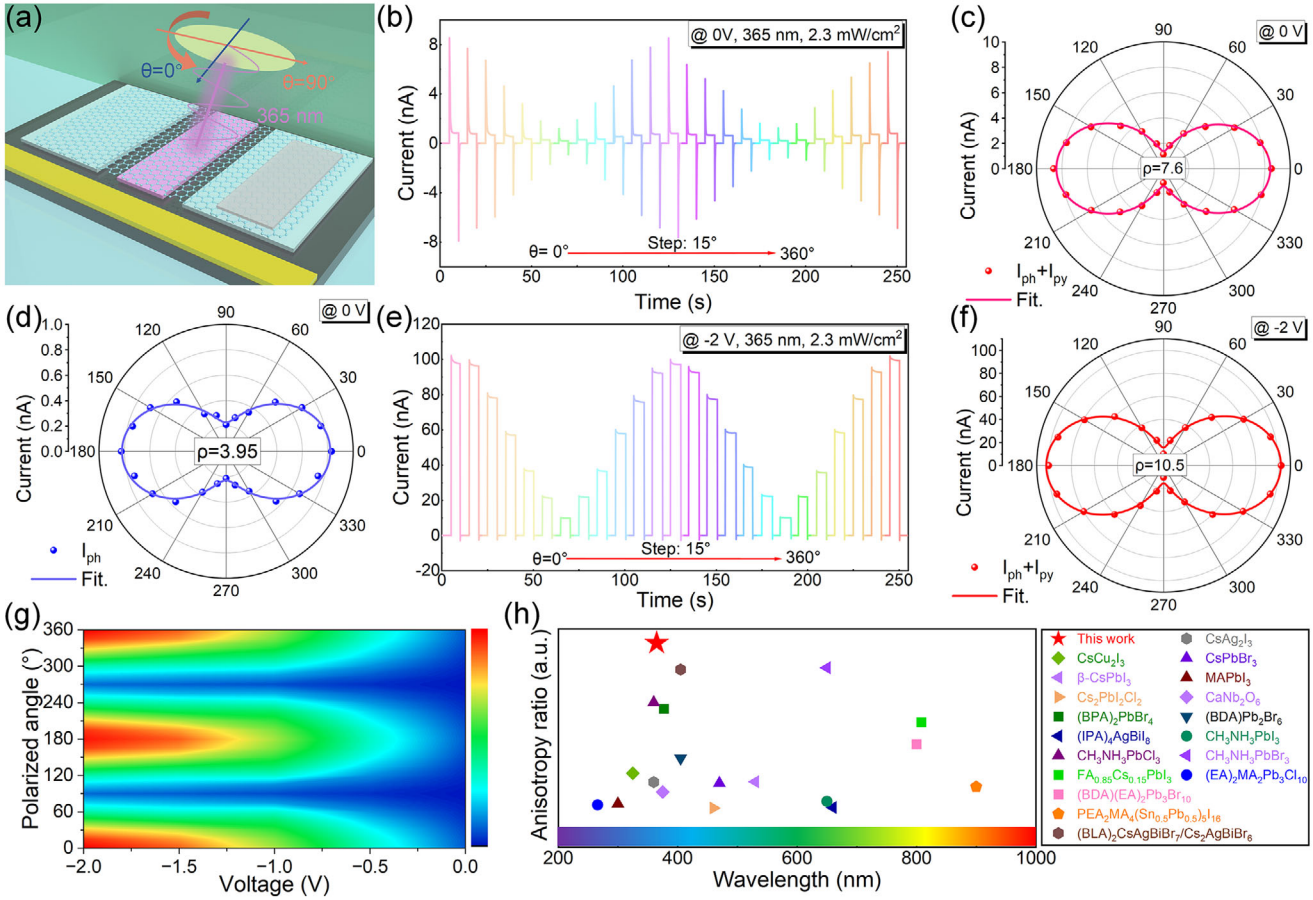
Polarization characteristics and performance comparison of the fabricated CsAg_2_I_3_/GaN vdWs heterojunction PD. (a) Schematic illustration of the polarization‐sensitive photodetection. (b) Time‐resolved photoresponse at 0 V bias under 365 nm LPL irradiation. Polar diagram of (c) *I*_ph+py_, and (d) *I*_ph_ under 0 V bias with the corresponding anisotropic ratios 7.6 and 3.95, respectively. (e) Time‐resolved photoresponse at −2 V bias. (f) Polar diagram of polarized responsivity under −2 V bias with the anisotropic ratio 10.5. (g) *I*_ph+py_ mapping at different voltages under illumination at 365 nm. (h) Summarized the anisotropic ratio of some representative perovskite‐based PDs.

To further investigate the regulatory role of the pyro‐phototronic effect in linearly polarized light detection, the polarization‐sensitive detection performance of the designed CsAg_2_I_3_/GaN vdWs heterojunction PD was studied by adjusting the incident light polarization angle using a half‐wave plate, as shown in Figure 5a. The dynamic photoresponse of the polarization‐sensitive detector can be continuously recorded by alternately switching the 365 nm laser on/off while synchronously rotating the half‐wave plate angle. Under zero‐bias conditions, the transient pyroelectric current (*I*_py_) and photocurrent (*I*_ph_) intensity were observed to exhibit the same periodic variations as the half‐wave plate was rotated, as depicted in Figure 5b. The *I*_ph+py_ intensity reaches its minimum at polarization angles of 90° and 270°, and its maximum at 0°, 180°, and 360°, respectively. As presented in the polarization plot (Figure 5c), the anisotropy ratio (*I*_max_/*I*_min_) reaches approximately 7.6, significantly exceeding the value of 3.95 (Figure 5d) observed for the pure photocurrent. The results demonstrate that the pyro‐phototronic effect can significantly modulate the polarization ratio, which is the first observation of this phenomenon in all‐inorganic perovskites.

Furthermore, by applying an external bias of −2 V, the dynamic photocurrent, as depicted in Figure 5e, reveals that although the *I*_py_ is still present, it is no longer the primary regulator of the polarization photocurrent due to the enhancement of the *I*_ph_. The corresponding polarization plot is shown in Figure 5f, where the anisotropy ratio has been enhanced to 10.5. The polarization patterns under different bias voltages are presented in Figure S22. Figure 5g shows polarization‐dependent current (*I*_ph+py_) mapping at different voltages under illumination at 365 nm. This demonstrates that the modulation of the device from self‐driven to −2 V bias voltage results in an astonishingly high anisotropy ratio for Ag‐based perovskite PDs. This is because when the polarization angle is parallel to the built‐in electric field, the photogenerated carriers have the maximum momentum in the direction of the built‐in electric field [35]. This effectively separates the generated electron–hole pairs and reduces their recombination. Under an externally applied bias voltage, the enhanced electric field accelerates the separation of charge carriers, thereby accentuating the anisotropy of the photocurrent, as shown in Figure S23. In addition, the van der Waals heterojunction device achieves a higher dichroic ratio compared to the MSM structured device, attributable to the following factors: (1) The heterojunction significantly amplifies the photocurrent differences across different polarization directions by leveraging the polarization‐dependent carrier separation efficiency provided by the built‐in electric field of the p‐n junction [36]. (2) Due to differential light absorption at varying polarization states, stronger light absorption generates a greater number of thermoelectrons, thereby producing a more pronounced pyroelectric signal and enhancing the dichroic ratio. Figure 5h depicts, compared to various mainstream polarization‐sensitive PDs, the high anisotropy of the CsAg_2_I_3_/GaN vdWs heterojunction PD places it at the forefront in the field of UV photodetection [10, 37, 38, 39, 40, 41, 42, 43, 44, 45, 46, 47, 48, 49, 50, 51, 52].

**FIGURE 6:**
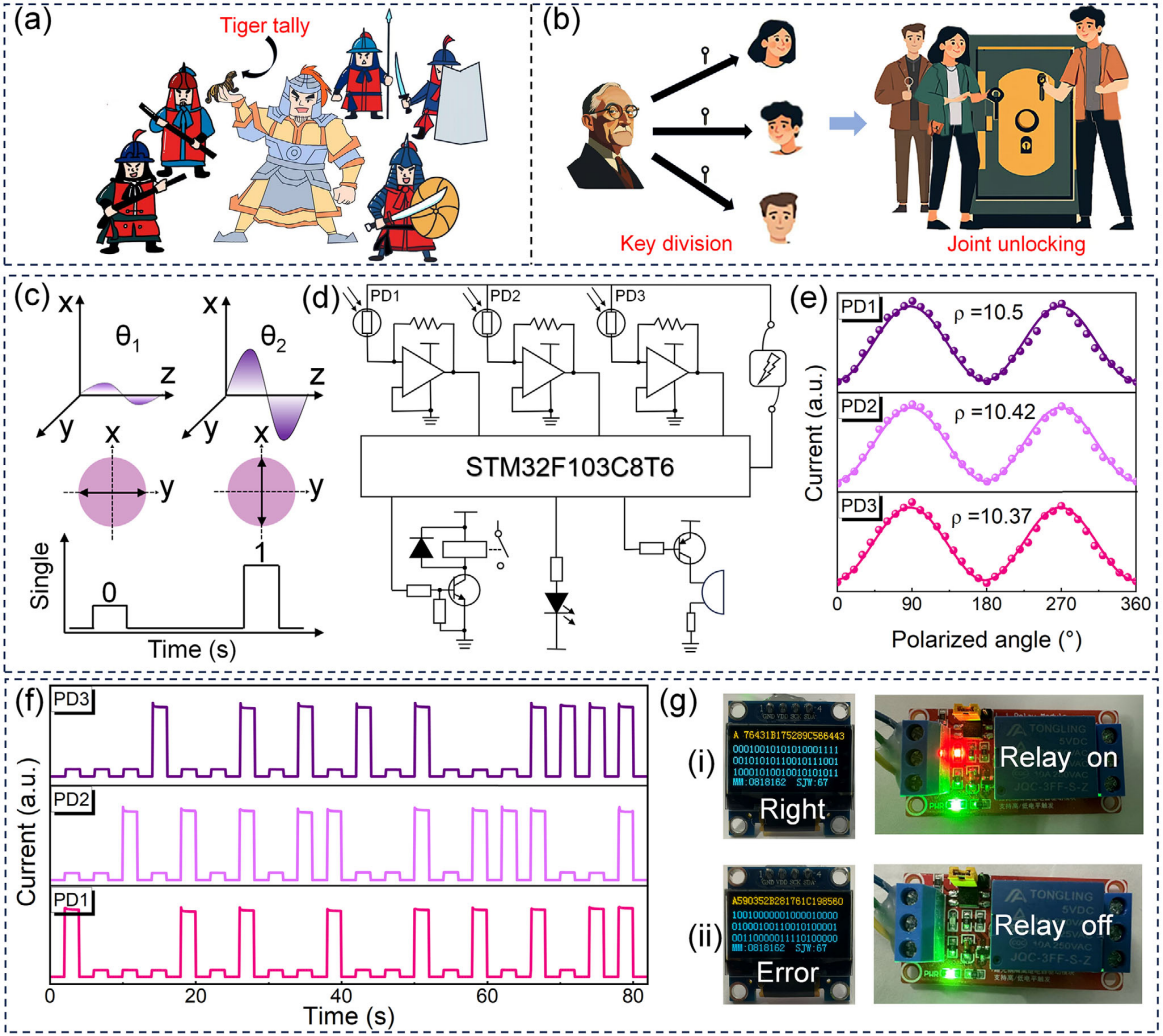
Application of key splitting based on multiple PDs with a high anisotropic ratio. (a) A complete tiger tally commanding soldier. (b) Distributed key management in banks. (c) Two polarized signal pulses at the transmitting end and two corresponding photoelectric signals at the receiving end. (d) The circuit schematic diagram of the key splitting system. (e) The polarization current characteristics of three PDs. (f) The photoelectric signals generated by PDs. (g) The OLED display module and physical display.

The principle of split‐key secret sharing, historically exemplified by the ancient Chinese tiger tally system (Figure 6a) and contemporary bank vault access protocols (Figure 6b), has persistently safeguarded valuable assets. However, implementing this cryptographic tenet in optoelectronic hardware remains a formidable challenge. Here, we demonstrate a high‐performance self‐powered UV detector with intrinsic polarization sensitivity, whose exceptional performance enables practical, secure, and energy‐efficient optical communication systems. This device thus embodies the convergence of historical wisdom and modern photonics. Capitalizing on its capabilities, we further devise a cryptographic scheme that leverages the polarization state of 365 nm light for secure key distribution (Figure 6c). Figure 6d shows the circuit architecture for implementing secret key splitting. At the transmitter end, three polarized light beams of identical intensity serve as pulsed light sources. Distinct photocurrents are generated based on the polarization sensitivity of three PDs. These three currents are converted into voltage signals and then fed into a microcontroller system. Through analog‐to‐digital conversion (ADC), the voltage signals are transformed into digital signals (0 and 1). Leveraging the system's memory storage capability, the optical signals are converted into digital codes. If the summation of these codes matches the pre‐stored password, the relay activates, and the LED module emits red light flashes to indicate correct password verification. Conversely, if the summation diverges from the pre‐stored password, the relay remains inactive while the buzzer system triggers an alarm.

The system employs three PDs with nearly identical polarization sensitivity, as illustrated by the polarization photocurrent shown in Figure 6e. This phenomenon not only demonstrates the excellent uniformity of the fabricated devices but also highlights the reliable accuracy inherent in the system design. When illuminated with pulsed light at different polarization angles, the corresponding photocurrents of the three PDs are sequentially displayed in Figure 6f. The represented password, shown in Figure 6g(i), sums to the preset code “0618162”, with the illumination of the red light indicating relay activation and successful authentication. Conversely, when the photocurrent patterns deviate from those in Figure 6f, the relay remains inactive due to password mismatch, as demonstrated in Figure 6g(ii). This operational logic validates the system's authentication mechanism: correct current sequences activate the relay through code validation, while incorrect patterns ensure system security by blocking access. Due to its excellent polarization detection capability for pulsed signals and stable response, this polarization‐sensitive PD is expected to demonstrate significant potential for broad information encryption applications, ranging from civilian to military domains.

## 3 Conclusions

In summary, this work demonstrates a breakthrough in self‐powered UV photodetection by synergistically integrating the pyro‐phototronic effect with a vdWs heterojunction architecture. The CVD‐grown non‐centrosymmetric CsAg_2_I_3_ SCs exhibit a 3.38 eV bandgap, exceptional in‐plane anisotropy, strong nonlinear optical response, and pronounced pyroelectricity, overcoming the conventional trade‐off between structural asymmetry, wide bandgap, and stability. The constructed CsAg_2_I_3_/GaN vdWs heterojunction achieves an atomically sharp interface with ultralow pA‐level dark current and enables a comprehensively augmented photoresponse through the pyro‐phototronic coupling. Remarkably, the device delivers state‐of‐the‐art performance: a high responsivity of 0.28 A/W, a specific detectivity of 1.7 × 10^12^ Jones, ultrafast response speeds of 16/21 µs, and an unprecedented polarization ratio of 10.5 under bias, surpassing most conventional wide‐bandgap semiconductor‐based detectors. Furthermore, we successfully implemented this polarization‐sensitive detector in an information‐splitting encryption system, validating its practical potential for secure optical communication. This study not only establishes a new paradigm for pyro‐phototronic‐enhanced polarized‐light detection but also provides a scalable material platform and device blueprint for next‐generation intelligent, self‐powered optoelectronic systems in advanced security and sensing applications.

## 4 Experimental Section

### 4.1 Crystal Growth

A novel chemical vapor deposition (CVD) method was employed to grow high‐quality, low‐dimensional ribbon‐like CsAg_2_I_3_ monocrystalline structures on SiO_2_/Si substrates. In the synthesis, CsI (99.9%) and AgI (99.9%) were manually ground and thoroughly mixed in a 1:2 stoichiometric ratio to serve as the reaction precursors. The precursor mixture was placed in a corundum boat, which was positioned in the upstream region of a tube furnace. The SiO_2_/Si substrate was placed in the downstream section as the growth substrate. Argon gas (∼ 40 sccm) was used as the carrier gas to facilitate the transport of gaseous cesium, silver, and iodine species. The pressure was maintained at 50 Pa throughout the sample preparation process. After the tube furnace naturally cooled to room temperature, low‐dimensional CsAg_2_I_3_ samples were obtained on the substrate. By varying the reaction conditions, the product of individual CsAg_2_I_3_ SCs with well‐defined length, width, and thickness could be achieved.

### 4.2 Characterizations

Comprehensive characterizations of the structure and photoelectric properties of the CsAg_2_I_3_ samples were carried out. The morphology of the samples was evaluated using scanning electron microscopy (SEM, TESCAN LYRA3 GM) and atomic force microscopy (AFM, Bruker Dimension Edge). The elemental distribution of CsAg_2_I_3_ SCs was analyzed using energy‐dispersive X‐ray spectroscopy (EDS) analysis. The crystal structure of CsAg_2_I_3_ SCs was investigated using high‐resolution transmission electron microscopy (HRTEM, Talos F200s). The X‐ray diffractogram of Rb_2_AgI_3_ SCs was obtained via X‐ray diffraction (XRD, X'Pert3 Power). Chemical state identification was performed using X‐ray photoelectron spectroscopy (XPS, Kratos AXIS Supra). Micro‐UV–vis absorption spectroscopy was performed by using the Metatest ScanPro Advance system. PL and PLE measurements of CsAg_2_I_3_ SCs were measured by using a fluorescence spectrometer (Hitachi F7000). The photoelectrical properties and photoresponse performances of the PD were characterized by using a scanning photoelectric test system (MStarter 200).

### 4.3 Calculation Details

All model optimizations were conducted through the solution of the Kohn‐Sham equation. The electron density function, denoted as *ρ*(r), which delineates the system's state, was determined. Subsequently, the adsorption energy, charge transfer, and density of states of the adsorption system were computed based on this function. The Kohn‐Sham equation solution process involves a self‐consistent operation, wherein the convergence criterion for single‐atom energy within the self‐consistent field was established at 1 × 10^−5^ eV. In selecting the exchange‐correlation functional, the widely adopted generalized gradient approximation was chosen. Specifically, the Perdew–Burke–Ernzerhof exchange‐correlation functional was employed to calculate the exchange‐correlation energy. Atomic orbitals were computed utilizing the double numeric with polarization basis set. The Brillouin zone was discretized into a 3 × 3 × 1 *k*‐point mesh. For the self‐consistent field calculations, the maximum energy convergence criterion of the system was set at 1 × 10^−5^ Ha. The convergence criterion for the interaction force between atoms was defined as 0.02 Ha·nm^−1^, and the convergence criterion for the maximum displacement between atoms was set at 0.0005 nm. Structure optimization was deemed complete upon fulfillment of the aforementioned convergence criteria.

## Conflicts of Interest

The authors declare no conflicts of interest.

## Supporting information




**Supporting File**: advs74896‐sup‐0001‐SuppMat.docx.

## Data Availability

The data that support the findings of this study are available from the corresponding author upon reasonable request.
