# Supplementary material for: Synergistic Pyro‐Phototronics and Structural Anisotropy in CsAg2I3/GaN Heterostructures for High‐Performance Polarization‐Sensitive UV Photodetectors
Source: Adv Sci (Weinh). 2026 Mar 18;13(31):e74896. doi: 10.1002/advs.74896 (PMC13588079; doi:10.1002/advs.74896)
Supplement: Supplementary file 1 [file ADVS-13-e74896-s001.docx]

**Supporting Information**

Synergistic Pyro-Phototronics and Structural Anisotropy in CsAg_2_I_3_/GaN Heterostructures for High-Performance Polarization-Sensitive UV Photodetectors

Yalin Zhai, Siqi Wang, Peng Guo, Songling Feng, Peng Wan, Jilong Tang, Caixia Kan, Hui Li,* Daning Shi,* and Mingming Jiang*

Y. Zhai, P. Guo, S. Feng, P. Wan, C. Kan, D. Shi and M. Jiang

College of Physics

MIIT Key Laboratory of Aerospace Information Sensing and Physics

Key Laboratory for Intelligent Nano Materials and Devices

Nanjing University of Aeronautics and Astronautics

No. 29, Jiangjun Road, Nanjing Jiangsu Province, 211106, P. R. China.

E-mail: [shi@nuaa.edu.cn](mailto:shi@nuaa.edu.cn), https://orcid.org/0009-0003-4224-1398 (D. Shi); mmjiang@nuaa.edu.cn (M. Jiang)

S. Wang, H. Li

Jilin Key Laboratory of Solid-State Laser Technology and Application

School of Physics, Changchun University of Science and Technology

No.7089, Weixing Road, Changchun, Jilin Province, 130022, China

E-mail: [huili@cust.edu.cn](mailto:huili@cust.edu.cn), https://orcid.org/0000-0002-2665-979X (H. Li)

J. Tang

State Key Laboratory of High Power Semiconductor Laser

Changchun University of Science and Technology.

No.7089, Weixing Road, Changchun, Jilin Province, 130022, China

**Figure S1.** Sketch of the layered-like structure of CsAg_2_I_3_.

**Figure S2.** Width distribution profiles under different reaction conditions.

**Figure S3.** SEM images of ribbon-like CsAg_2_I_3_ single crystals (SCs) with varying widths.

**Figure S4.** EDS analysis of CVD-synthesized CsAg_2_I_3_ monocrystalline samples.

**Figure S5.** High-resolution transmission electron microscopy image (HRTEM) of the SC. No discernible defects were observed, indicating high crystallinity of the sample. The lattice fringe spacing is 0.857 nm, corresponding to the lattice space of (110) planes of orthorhombic CsAg_2_I_3_, which is consistent with the results of SCXRD.

**Figure S6.** The XRD pattern of the CsAg_2_I_3_ SC firmly demonstrates that the crystals belong to the orthorhombic structure of CsAg_2_I_3_ (PDF-00-052-1049). In addition, the XRD pattern shows four sharp peaks centered at 10.29°, 20.64°, 31.15°, and 41.94°, which are assigned respectively to (110), (220), (330), and (440) crystal planes. These highly oriented diffraction peaks also match well with the HRTEM spectrum, indicating that the CVD-grown CsAg_2_I_3_ samples exhibit phase pure with a high crystalline quality.

**Figure S7.** XPS survey spectrum of a) cesium and b) iodine in CsAg_2_I_3_ SCs. Cs and I core-level spectra show the 3d doublets with the binding energy of Cs 3d (724.5 eV, 738.5 eV) and I 3d (619.1 eV, 630.6 eV), which are consistent with Cs^+^ and I^−^, respectively.

**Figure S8.** The band structure and corresponding density of state (DOS) of CsAg_2_I_3_ obtained through first-principles calculations.

**Figure S9.** Time-resolved PL spectrum with the fitting curve of CsAg_2_I_3_.

**Figure S10.** Schematic diagram of testing method.

**Figure S11.** Thermal image of a) glass under dark, b) glass under UV illumination, c) sample/glass under dark, d) sample/glass under UV illumination, and e)-l) sample/glass after UV illumination is switch off.

**Figure S12.** Angle-resolved polarized SHG spectra of CsAg_2_I_3_ SCs in the parallel a) and perpendicular b) polarization configurations.

**Figure S13.** Schematic of the polarization absorption test setup.

**Figure S14.** Performance Characterization of MSM-Structured CsAg_2_I_3_ SCs Photodetectors. a) Wavelength-dependent photocurrent with a peak at 365 nm. b) *I-V* curves corresponding to different optical power densities under 365 nm light illumination. c) The fitted relationship between photocurrent versus illumination power density. d) *I–V* characteristic curves of the photodetector under darkness and 365 nm polarized light illumination. e) *I–T* characteristic curves of the photodetector at a fixed voltage of 5 V with respect to the different *θ*. f) Corresponding polar plots of polarized photocurrent.

**Figure S15:** Transmittance spectrum of the Gr monolayer.

**Figure S16.** *I-t* response of the CsAg_2_I_3_ SC/GaN vdWs heterojunction photodetector in the absence of external power, where visible or near infrared illumination is switched on and off periodically.

**Figure S17.** Energy band diagram of the self-powered CsAg_2_I_3_/GaN vdWs PD under illumination. Under UV illumination, a substantial number of electron-hole pairs are generated. Driven by the built-in electric field, electrons (holes) are collected by the Ni/Au (Gr) electrode and subsequently detected as current by the external circuit.

**Figure S18.** *I-t* response of the detector in the absence of external power, where UV illumination (*λ*=365 nm, 3 mW cm^−2^) is switched on and off periodically.

**Figure S19.** Current as functions of power densities.

**Figure S20.** The presence of the pyro-phototronic effect results in significant enhancement for *R*, *D**, and *EQE*.

**Figure S21:** *R* and *EQE* of our detector after storage for 15 weeks.

**Figure S22.** Current polarization plots under applied biases of −0.5 V, −1 V, and −1.5 V.

**Figure S23.** The zero-degree polarization angle is collinear with the built-in electric field vector.

# Table S1. Elaboration of experimental variables.

| **Condition** | **Precursor mass (g)** | **Heating rate (℃/min)** | **Reaction temperature (℃)** | **Holding time (min)** |
| --- | --- | --- | --- | --- |
| **1** | 2.0 | 15 | 550 | 30 |
| **2** | 2.0 | 15 | 550 | 35 |
| **3** | 2.2 | 15 | 550 | 35 |
| **4** | 2.2 | 20 | 565 | 40 |
| **5** | 2.4 | 20 | 580 | 40 |

**Table S2:** Comprehensive comparison of heterojunction photodetectors based on CsAg_2_I_3_ grown by different processes.

| Preparation methods | *R* (mA/W) | *D** (Jones) | *EQE* (%) | Response speeds | Dichroic ratio | Ref. |
| --- | --- | --- | --- | --- | --- | --- |
| spatial confinement growth | 48.5 | 1.13×10^11^ | 16.5 | 6.5/224 μs | - | ^[1]^ |
| space-confined | 35 | 1.8×10^12^ | - | 17/18 ms | - | ^[2]^ |
| space-conﬁned | 26.5 | 2.09×10^8^ | - | 258.43/289.32 ms | - | ^[3]^ |
| pyridine additive-assisted one-step solution | 32 | 1.2×10^11^ | 12.3 | 47/74 μs | - | ^[4]^ |
| CVD | 280 | 1.7×10^12^ | 95.3 | 16/21 μs | 10.5 | This work |

References

[1]	W. Sun, S. Liu, C. Wang, X. Zu, S. Li, X. Xiang, *J. Phys. Chem. Lett.* **2024**, 15, 2359.

[2]	X. Wang, P. Gui, X. Wei, Z. Wang, T. Hu, L. Yang, S. Wang, Z. Huang, *IEEE Electron Device Lett.* **2024**, 45, 1488.

[3]	M. Zhang, J. Yan, J. Ma, X. Wang, T. Li, H. Jiang, X. Ji, M. Wang, R. Yang, Z. Liu, *Adv. Opt. Mater.* **2023**, 11, 2203054.

[4]	Z. Hu, B. Zhang, F. Zhang, X. Guo, Z. Lin, J. Zhang, Y. Hao, J. Chang, *Sci. China Mater.* **2023**, 66, 3629.
